# Supplementary figures and images for: Natrix: a Snakemake-based workflow for processing, clustering, and taxonomically assigning amplicon sequencing reads
Source: BMC Bioinformatics. 2020 Nov 16;21:526. doi: 10.1186/s12859-020-03852-4 (PMC7667751; doi:10.1186/s12859-020-03852-4)

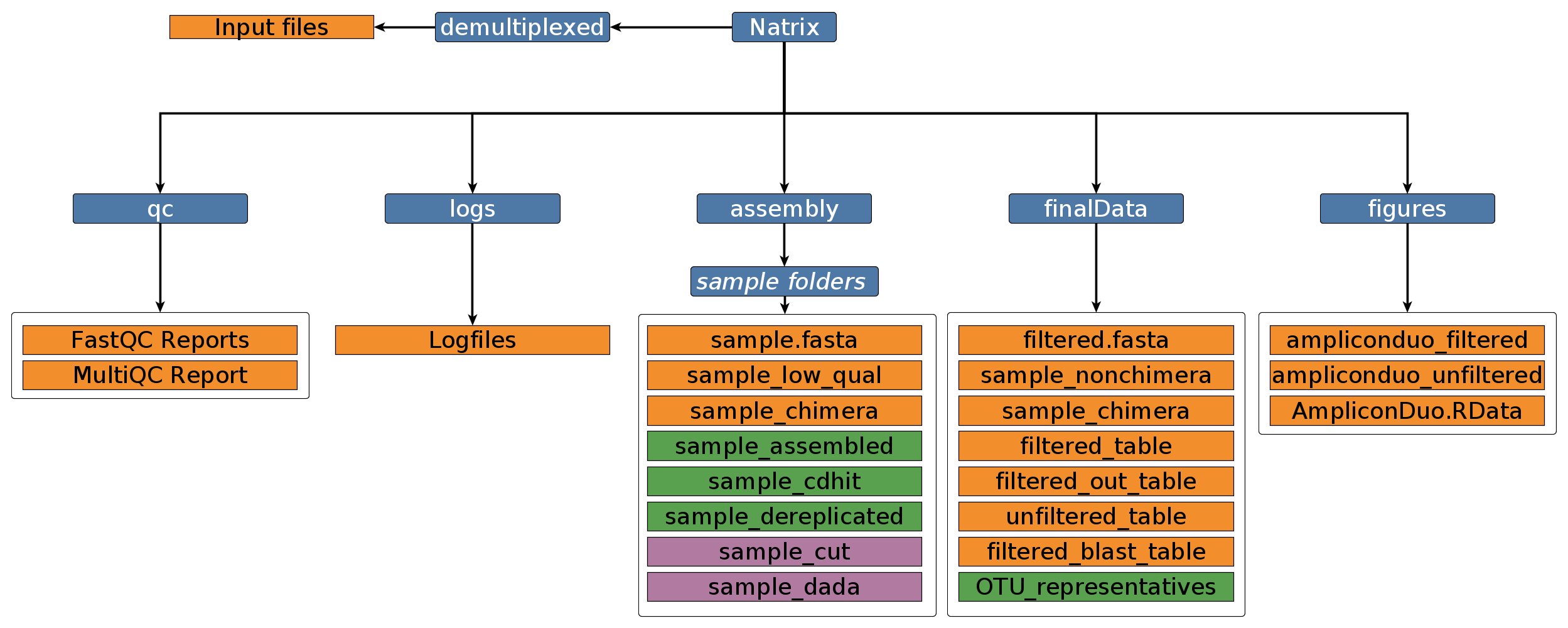

Supplement: Supplementary file 2 — Additional file 2. Output file hierarchy. Output file hierarchy in PNG format, blue nodes represent folders, orange nodes represent files that are created in both variants of the workflow, green nodes are files exclusive to the OTU variant and purple nodes are files exclusive to the ASV variant of the workflow. [file 12859_2020_3852_MOESM2_ESM.png]
